# Analysis of drug-induced and spontaneous cardioversions reveals similar patterns leading to termination of atrial fibrillation

**DOI:** 10.3389/fphys.2024.1399037

**Published:** 2024-07-18

**Authors:** Arne van Hunnik, Vladimír Sobota, Stef Zeemering, Dragan Opacic, Billy Scaf, Elisa D’Alessandro, Karel Oyaert, Marion Kuiper, Jonas G. Diness, Ulrik S. Sørensen, James T. Milnes, Marcel A. G. van der Heyden, Thomas Jespersen, Ulrich Schotten, Sander Verheule

**Affiliations:** ^1^ Department of Physiology, Cardiovascular Research Institute Maastricht, Maastricht, Netherlands; ^2^ IHU-LIRYC, Electrophysiology and Heart Modeling Institute, Fondation Bordeaux Université, Bordeaux, France; ^3^ Institute de Mathématiques de Bordeaux, University of Bordeaux, Talence, France; ^4^ Acesion Pharma, Copenhagen, Denmark; ^5^ Xention Ltd., Cambridge, United Kingdom; ^6^ Department of Medical Physiology, University Medical Center Utrecht, Utrecht, Netherlands; ^7^ Department of Biomedical Sciences, University of Copenhagen, Copenhagen, Denmark

**Keywords:** antiarrhythmic drugs, atrial anatomy, atrial fibrillation, Bachmann’s bundle, cardioversion

## Abstract

**Introduction:**

The mechanisms leading to the conversion of atrial fibrillation (AF) to sinus rhythm are poorly understood. This study describes the dynamic behavior of electrophysiological parameters and conduction patterns leading to spontaneous and pharmacological AF termination.

**Methods:**

Five independent groups of goats were investigated: (1) spontaneous termination of AF, and drug-induced terminations of AF by various potassium channel inhibitors: (2) AP14145, (3) PA-6, (4) XAF-1407, and (5) vernakalant. Bi-atrial contact mapping was performed during an open chest surgery and intervals with continuous and discrete atrial activity were determined. AF cycle length (AFCL), conduction velocity and path length were calculated for each interval, and the final conduction pattern preceding AF termination was evaluated.

**Results:**

AF termination was preceded by a sudden episode of discrete activity both in the presence and absence of an antiarrhythmic drug. This episode was accompanied by substantial increases in AFCL and conduction velocity, resulting in prolongation of path length. In 77% ± 4% of all terminations the conduction pattern preceding AF termination involved medial to lateral conduction along Bachmann’s bundle into both atria, followed by anterior to posterior conduction. This finding suggests conduction block in the interatrial septum and/or pulmonary vein area as final step of AF termination.

**Conclusion:**

AF termination is preceded by an increased organization of fibrillatory conduction. The termination itself is a sudden process with a critical role for the interplay between spatiotemporal organization and anatomical structure.

## 1 Introduction

Mechanisms underlying perpetuation of atrial fibrillation (AF) have been extensively investigated over the last decades. Several concepts are recognized as important contributors, including anatomical and functional re-entry, ectopic activity, multiple wavelets and endo-epicardial dissociation (Schotten et al., 2011). However, the mechanisms of conversion of AF to sinus rhythm (SR) are not well understood. During this transition, mechanisms that drive AF disappear or lose their ability to maintain the arrhythmia. In-depth understanding of AF terminating mechanisms might help to refine AF therapy.

Several studies have investigated the changes in AF properties during the conversion of persistent AF to SR under the influence of antiarrhythmic drugs (AADs) (Wijffels et al., 2000; Workman et al., 2011). AF cycle length (AFCL) prolongation is the most consistent observation, irrespective of the class of the AAD (Workman et al., 2011). Prolongation of AFCL often goes along with an organization of the fibrillation pattern and a reduced number of wave fronts (Wang et al., 1993; Ji et al., 2017; Sobota et al., 2021; van Hunnik et al., 2021). However, these studies generally used “steady state” time points for the characterization of AF, while AF is a highly dynamic process (van Hunnik et al., 2018). Some studies suggest that changes in AF dynamics take place just prior to AF termination. For instance, AF termination induced by some AADs was associated with an additional sudden increase in AFCL just seconds before AF termination (Gatta et al., 2021; Sobota et al., 2021). Also, for some AADs AF did not organize in a dose-dependent manner but only exhibited organization of atrial conduction in the final seconds before AF termination (Gatta et al., 2021; Sobota et al., 2021).

Considering that a sudden change in AF properties preceded the arrhythmia termination in several AAD studies, we hypothesized that shared mechanisms may contribute to the transition from AF to SR. Therefore, we investigated AF termination under a variety of conditions. Studies of spontaneous termination of acutely-induced AF and maintained (3–4 weeks) AF were included. In addition, we included studies of termination of maintained AF, induced by four different AADs with a wide range of ion channel targets. Using a goat model of AF, we performed synchronous bi-atrial high-density electrical contact mapping to construct conduction patterns. Dynamic behavior of spatiotemporal organization and electrophysiological parameters were analyzed to capture the transition from AF to SR.

## 2 Methods

### 2.1 Study design

Animal care and management complied with the guidelines from Directive 2010/63/EU of the European Parliament. This study consists of five independent experimental groups.

(I) Spontaneous termination of acute AF (n = 8),
(II) Small conductance calcium-activated potassium current (I_SK_) inhibition by AP14145 (n = 8),
(III) Inward rectifier potassium current (I_K1_) inhibition by PA-6 (n = 7),
(IV) Sodium and multiple potassium currents inhibition by vernakalant (n = 8),
(V) Acetylcholine-activated potassium current (I_K,ACh_) inhibition by XAF-1407 (n = 9).

Some results from the groups II-V have been published previously (Ji et al., 2017; van Hunnik et al., 2018; 2021; Gatta et al., 2021; Sobota et al., 2021).

### 2.2 Animal instrumentation and AF induction

**FIGURE 1:**
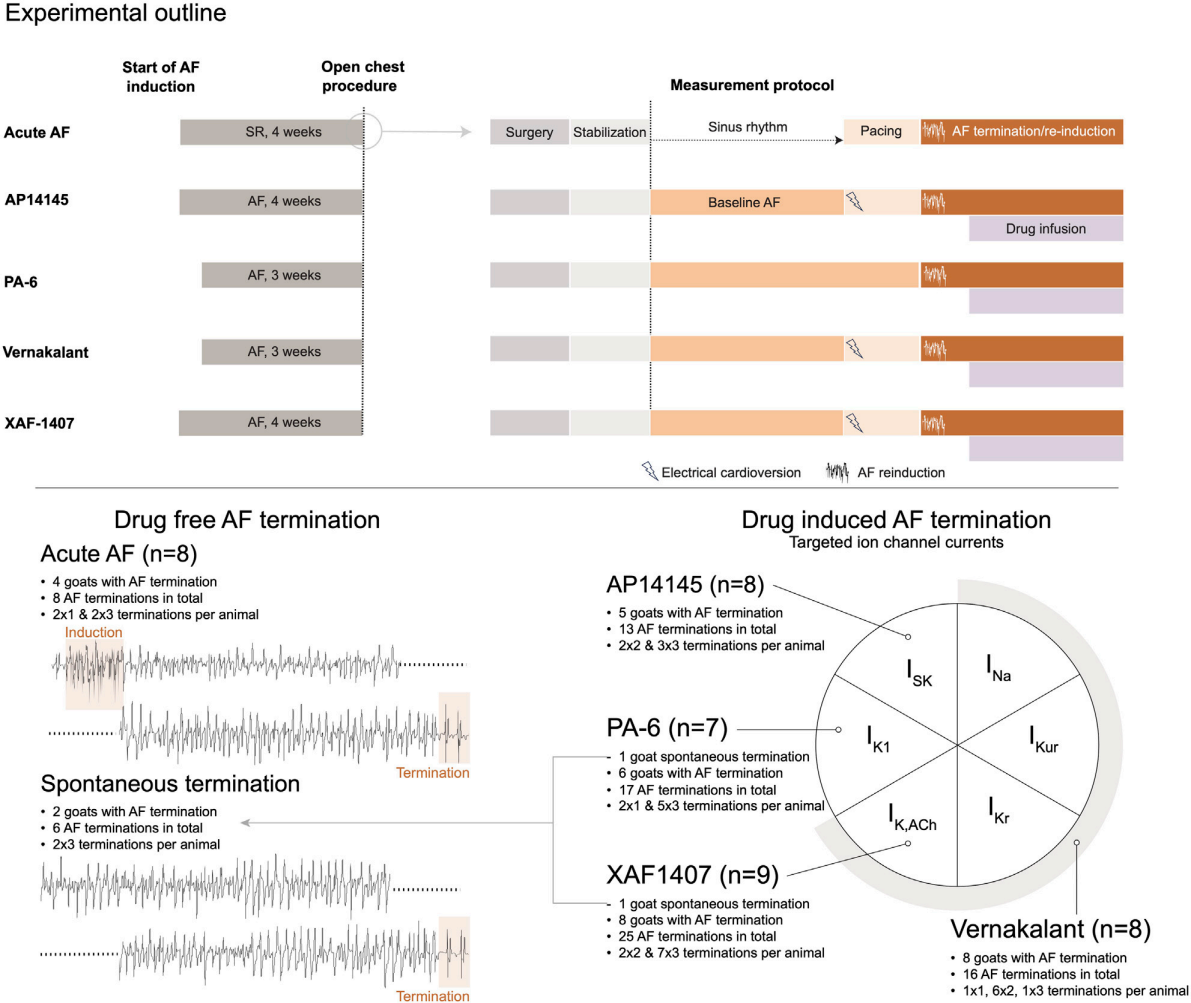
Study design. At the top a schematic outline of the experimental procedures is provided for each group. The left panel represents the chronic phase of AF induction or sham. The open chest procedure, indicated by a circle, is presented in more detail in the right panel. This panel presents the procedural steps in chronology from surgery to baseline AF and AF termination measurements for each group. At the bottom, a graphical representation of the distinct experimental groups is provided, displaying the number of animals per group, the number of animals with at least one AF termination, and the overall number of AF terminations per group. The diagram on the right side depicts the ion currents inhibited by the antiarrhythmic drugs. AF, atrial fibrillation; SR, sinus rhythm.

A left-sided thoracotomy was performed under general anesthesia (sufentanil 6 μg/kg/h IV and propofol 5–10 mg/kg/h IV) to implant a custom-built patch of electrodes on the pericardium overlying the left atrium for atrial rhythm monitoring. One electrode pair was connected to a subcutaneous neurostimulator (Itrel^®^, Medtronic, Minneapolis, Minnesota, United States). The animals received post-operative treatment with antibiotics (ampicillin, 10 mg/kg IM, three times a day, first 2 days after the surgery) and analgesia with buprenofine (10 μg/kg IM twice a day, first day after the surgery) and carprofen (2–4 mg/kg SC, first 3 days after surgery). After a recovery period of 2–3 weeks, AF was induced and maintained in groups II-V for 3–4 weeks by burst pacing (50 Hz, 10 V, 1 s on 1 s off). The Acute AF group (I) did not undergo AF induction but remained in sinus rhythm over the same period. Schematic outline of the experimental procedures for each group is provided in Figure 1.

### 2.3 Sacrifice protocol and data acquisition

An open chest study under general anesthesia (sufentanil 6 μg/kg/h, propofol 10 mg/kg/h and rocuronium 0.3 mg/kg/h) was performed in all groups. A left-sided thoracotomy allowed placement of two octagonal mapping arrays (248 electrodes each, interelectrode distance 2.4 mm), one on the right atrial (RA) and one on the left atrial (LA) wall. On the RA the electrode array covered the epicardium from the appendage to the inter-caval junction. On the LA the electrode array covered the epicardium from the appendage to the onset of the right pulmonary vein. The same position and orientation of the electrode arrays (with respect to the anatomical landmarks) was used in all animals. The arrays were kept in fixed position during the whole experiment by mechanical stabilizers. In the vernakalant group an additional rectangular electrode was placed on the Bachmann’s bundle (58 electrodes, interelectrode distance 5 mm).

Surgical instrumentation was followed by a stabilization period of 30 min without measurements or manipulations (Figure 1). Next, AF was recorded under baseline conditions. The animals in the Acute AF group were in SR at this point. In the other groups, AF was terminated after the baseline recordings by internal defibrillation (Physio-Control Lifepak 9B, Medtronic, Minneapolis, Minnesota, United States). Subsequently, S1S1 and S1S2 pacing protocols were performed, except for the PA-6 group (Figure 1). These data were published previously (Gatta et al., 2021; Sobota et al., 2021; van Hunnik et al., 2021) and are not included in this study.

After these measurements, AF was (re)induced in all groups by a 1-s burst of 50 Hz and unipolar electrograms were continuously recorded (sampling rate of 1 kHz, 16-bit resolution, bandpass filter of 0.56–408 Hz). In case a conversion to SR occurred, an automatic algorithm reinduced AF within one second. AF was maintained in this way for ≥20 min. The intravenous drug administration was initiated afterwards. The following drug infusion regimens were applied: AP14145 was infused at a rate of 20 mg/kg/h for 45 min; PA-6 was infused at a rate of 2.5 mg/kg over 10 min as a bolus, followed by a continuous infusion of 0.04 mg/kg/min; Vernakalant was infused at a rate of 3.7 mg/kg over 15 min as a bolus, followed by a continuous infusion of 2.0 mg/kg/min; XAF-1407 was infused at a rate of 0.3 mg/kg over 20 min, followed by a maintenance dose of 15 μg/kg/min and a subsequent dose of 3.0 mg/kg. Unipolar electrogram recording and rhythm monitoring were continued during the drug infusion phase over a period of at least 30 min to record up to 3 AF terminations. AF was reinitiated after each AF termination. At the end of the protocol the animals were euthanized by induction of ventricular fibrillation or by exsanguination.

### 2.4 Pre-processing of AF recordings

For baseline AF analysis, six consecutive 10-s intervals were analyzed. For the AF terminations analysis, the terminations of the first three AF episodes outlasting 10 s were used. From these terminations the electrograms capturing the last 10 s before AF termination were analyzed, including the last AF activation cycle. Ten second recordings were used because explorative analysis of the stability of AF properties in a previously published study (van Hunnik et al., 2018) demonstrated a high degree of stability within such time interval. The last AF cycle was discriminated from the first SR beat by distinctly different morphologies of atrial unipolar electrograms combined with a lack of a P-wave in the ECG.

Custom-made software developed in Matlab (The MathWorks, Inc., Natick, Massachusetts, United States) was used for identification of local activation times with a previously validated probabilistic annotation algorithm (Zeemering et al., 2012). In short, a unipolar electrogram exhibits RS morphology for a passing wavefront. The algorithm identifies all RS morphologies with widths of 5–50 ms. A probability density function of the AF cycle length was found by excluding 10% of the deflections with the lowest amplitudes. Then, a heuristic greedy algorithm used this probability density function to distinguish local activations and determine activation times. Conduction velocity (CV) was calculated using a plane fit through the central activation time and its immediate spatial neighbors (minimum 3, maximum 8), where the steepness of this plane corresponds proportionally to the velocity. To avoid bias during signal processing and pattern interpretation, the operator (AvH) was blinded to the study group assignment. The operator evaluated activation time videos in conjunction with unipolar electrograms to eliminate invalid activation detections due to artefacts or ventricular far-fields. The same operator evaluated the final conduction pattern before AF termination.

### 2.5 Analysis of dynamic properties of AF

**FIGURE 2:**
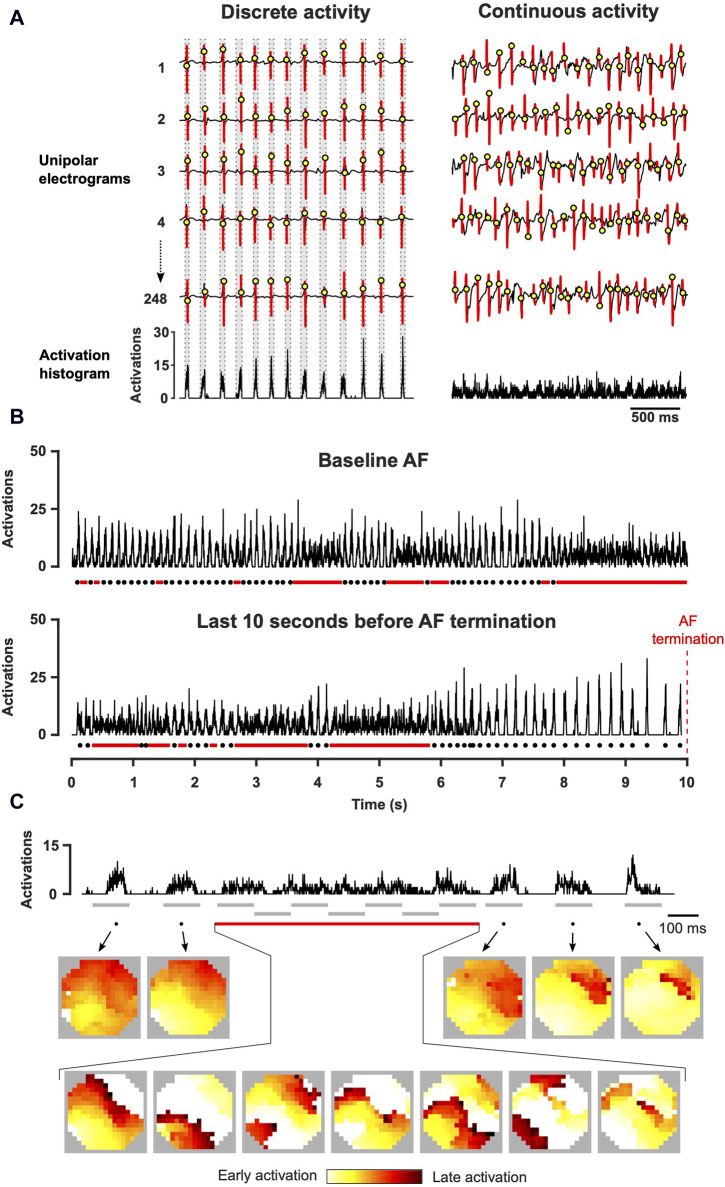
Assessment of spatiotemporal organization with the construction of activation time histograms. **(A)** Local atrial activations were determined from unipolar electrograms. Activation time histogram represents the summation of these time points for all channels in the electrode array. **(B)** Representative examples of activation time histograms acquired at baseline AF and before AF termination (right atrium, XAF-1407 group). Dots indicate cycles of discrete activity and lines continuous activity. **(C)** Activation time histogram and the corresponding conduction patterns (left atrium, AP14145 group). During discrete activity, one or a few fibrillatory wave fronts activated the mapped area but multiple waves were continuously present during continuous activity. The grey lines indicate the intervals depicted in the activation maps.

The examination of AF activation time videos indicated varying periods of low and high complexity, which often exhibited dynamic changes within seconds. For example, during one instance, the atrial surface demonstrated propagation of a couple of nearly synchronous wave fronts, rapidly activating the mapping area within a short time window. However, at a later point, multiple fragmented and highly dyssynchronous wave fronts emerged, leading to a sustained period of continuous activity. This dynamic behavior of global electrophysiological parameters was analyzed for each atrium. To capture the beat-to-beat behavior, we identified periods of low and high spatiotemporal organization during AF. Temporal histograms of local activation times were constructed by determining the sum of all activations within a mapping array in each time step. To reduce noise, the histograms were binned by two samples (i.e., 2 ms) instead of the possible 1 ms step. This approach allowed the construction of a plot that depicts the number of activations at a given time step. During discrete activity, the histogram exhibits a beat-like pattern, showing clear peaks surrounded by periods without electrical activation, while during continuous activity, the beat-like pattern has disappeared and presents a thick band with low amplitude peaks (Figure 2). Discrete activity was formalized as an activation of the entire mapping area in one sequence of activations lasting shorter than 120 ms (equivalent to ≥32 cm/s for the entire map). In the activation histogram, a period of activations lasting over 120 ms was considered to be continuous activity. Identification of these two states allowed us to characterize the dynamic changes in AF organization and to study the beat-to-beat electrophysiological properties during discrete activity, including AF cycle length (AFCL), conduction velocity (CV) and path length (defined as AFCL×CV). AFCL and CV were calculated for each electrode within the mapping array, and average values were calculated for each interval of discrete and continuous activity. Average values over longer time periods were obtained as weighted averages, with the durations of individual intervals of discrete and continuous activity being used as weights.

### 2.6 Analysis of conduction patterns

Activation patterns that preceded AF termination were visually inspected and assigned to several categories based on the type of atrial conduction. The site of termination of fibrillatory wave front(s) propagation during the final AF cycle was considered as the location where the fibrillation wave front entered but no new wave front exited from.

In the series of experiments in the vernakalant group, an additional electrode was present at the Bachmann’s bundle, allowing more extensive assessment of atrial conduction. In this dataset, propagation over the Bachmann’s bundle was always linked to propagation over the atrial walls with a starting point close to the Bachmann’s bundle. In the other experimental groups, a pattern with a starting point close to the Bachmann’s bundle was therefore considered to be a Bachmann’s bundle-associated pattern.

### 2.7 Statistics

Based on previous studies that assessed fibrillation episodes in atria and ventricles (Everett IV et al., 2005; Witt et al., 2018), we considered AF terminations as independent events. The changes in electrophysiological parameters between baseline and AF termination were tested with a paired *t*-test, and between the atria with an unpaired *t*-test. Non-normally distributed data were tested with a Mann-Whitney *U* test or Friedman’s test. The differences in the duration of final discrete activity between the atria were tested with a Wilcoxon test, and the differences in the duration of continuous and discrete activity with a Mann-Whitney *U* test. The inter-atrial gradient in cycle length was tested with a linear mixed-effects model using a diagonal covariance structure with time and atrium as fixed variables and animal identity as random variable. A *p*-value <0.05 was considered to be statistically significant.

## 3 Results

In the Acute AF group, four out of eight goats were eligible for analysis because only in these goats AF paroxysms ≥10 s occurred. No baseline (i.e., stable AF) recordings were acquired in the Acute AF group. AAD infusion caused AF termination in five out of eight goats in the AP14145 group, six out of seven goats in the PA-6 group, eight out of eight goats in the vernakalant group and in eight out of nine goats in the XAF-1407 group. In 2 animals (1 animal from the PA-6 group and one animal from the XAF-1407 group) AF terminated spontaneously after 3–4 weeks of AF, hence during the baseline phase. These recordings will be referred to as a separate group, termed *Spontaneous termination*. Up to 3 AF terminations per animal were included in the study. In total, 79 AF terminations in 33 goats were evaluated, as summarized in Figure 1.

### 3.1 Early electrophysiological effects of the various antiarrhythmic drugs

**FIGURE 3:**
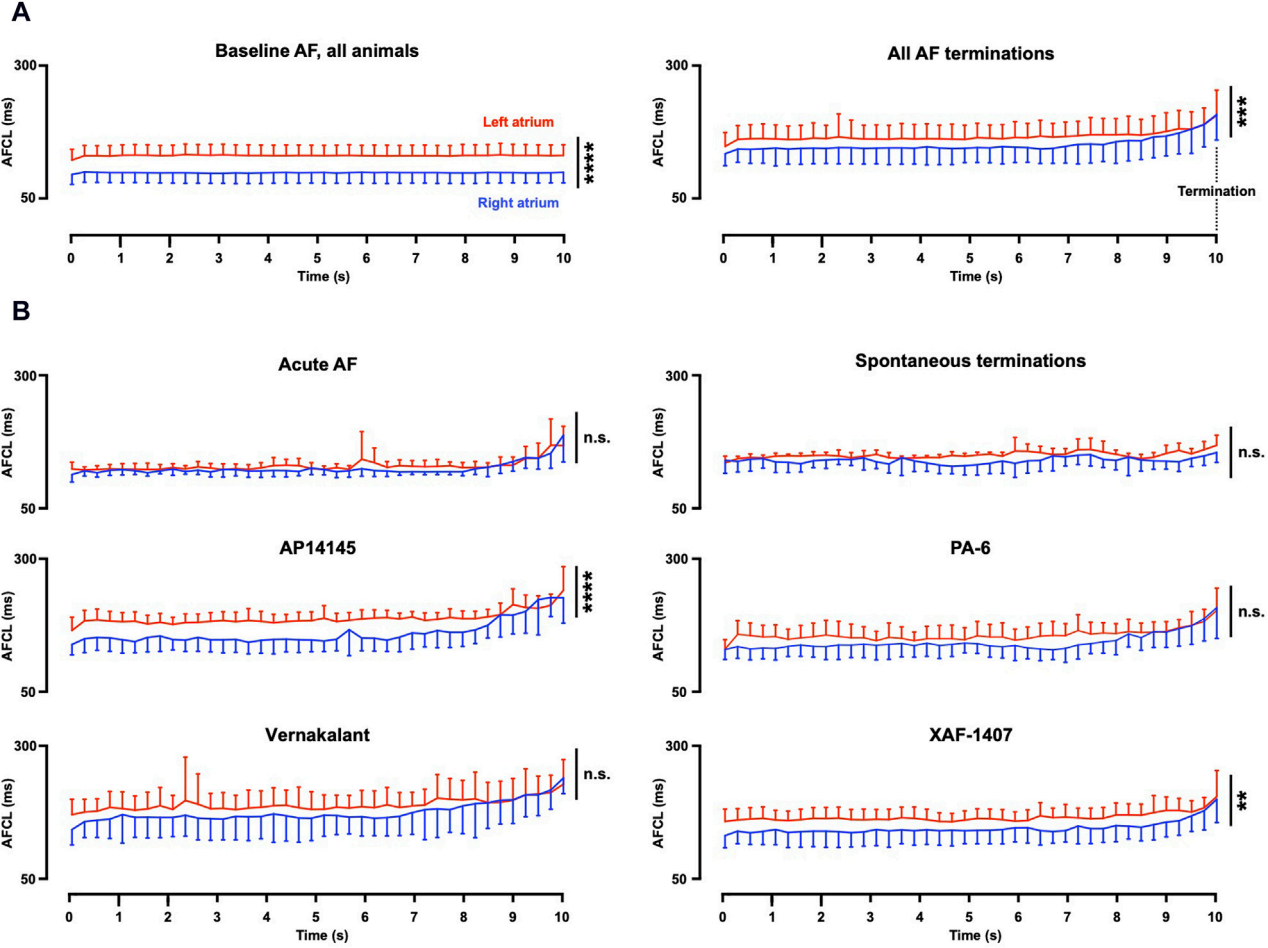
Inter-atrial AF cycle length gradient. **(A)** In all groups with stable baseline AF (27 animals), a gradient of the AF cycle length (AFCL) was present in baseline recordings. A somewhat smaller gradient was present during the 10 s before AF termination (33 animals, 79 terminations). This gradient gradually decreased with a full disappearance at 250 m before AF termination. **(B)** Individual group data revealed strong reduction of the AFCL gradient for Spontaneous termination, PA-6 and vernakalant. The AFCL gradient for AP14145 and XAF-1407 remained statistically different. Statistical analysis was performed with a mixed model. Terminations per group (animals per group); Acute AF n = 8 (4), Spontaneous termination n = 6 (2), AP14145 n = 13 (5), PA-6 n = 14 (6), Vernakalant n = 16 (8) and XAF-1407 n = 22 (8). AF, atrial fibrillation; **p < 0.01, ***p < 0.001, ****p < 0.0001, and n.s. Not significant.

Path length and AFCL were consistently prolonged at the last 10 s before AF termination in all AAD groups (Table 1), despite the broad range of ion current inhibiting profiles of the various AADs. A significant inter-atrial AFCL gradient was present at baseline, with a shorter AFCL in the RA (Table 1; Figure 3A). The inter-atrial AFCL gradient was somewhat attenuated during the 10 s before AF termination, it gradually decreased during the last 3 seconds and entirely disappeared in the final 250 m before AF termination. Individual group data showed that the AFCL gradient decreased earlier than 10 s prior to termination for vernakalant and PA-6 and was not present in the non-pharmacological terminations (Figure 3B). When present (AP14145, XAF-1407), the inter-atrial AFCL gradient gradually disappeared in the final seconds before termination.

**TABLE 1:** Electrophysiological changes from baseline to the final 10 s before AF termination. Statistical analysis of the differences between baseline and AF termination was performed with a paired *t*-test (**p < 0.01, ***p < 0.001). Statistical analysis of the differences between the left and right atrium was performed with an unpaired *t*-test (^#^ *p* < 0.05,^##^ *p* < 0.01,^###^ *p* < 0.001). Number of animals (and terminations) per group; All n = 33 (79), Acute AF n = 4 (8), Spontaneous termination n = 2 (6), AP14145 n = 5 (13), PA-6 n = 6 (14), Vernakalant n = 8 (16) and XAF-1407 n = 8 (22).

	Right atrium		Left atrium	
	Baseline	AF termination	Baseline	AF termination
AFCL (ms)				
All	99 ± 20	152 ± 28***	133 ± 18^###^	170 ± 27***^,###^
Acute AF	—	125 ± 7	—	126 ± 7
Spontaneous termination	—	135 ± 13	—	150 ± 4^#^
AP14145	111 ± 21	158 ± 17***	145 ± 11^#^	188 ± 12***^,###^
PA-6	111 ± 19	144 ± 22**	137 ± 24	158 ± 21
Vernakalant	84 ± 15	178 ± 33***	117 ± 13^###^	191 ± 26***
XAF-1407	93 ± 13	145 ± 22***	135 ± 14^###^	166 ± 16***^,###^
Conduction velocity (cm/s)				
All	52.8 ± 4.7	58.4 ± 8.2**	56 ± 4^#^	56.6 ± 8.3
Acute AF	—	61.1 ± 4.2	—	61.7 ± 5.7
Spontaneous termination	—	59.8 ± 5	—	62.3 ± 3.2
AP14145	56.9 ± 3.3	57.6 ± 6.4	56.1 ± 2.1	53.7 ± 6.2
PA-6	53.3 ± 2.5	56.9 ± 4.5	54.5 ± 4.5	57.4 ± 6.9
Vernakalant	46.9 ± 2.8	49.8 ± 5.4	56.4 ± 5.9^##^	50.3 ± 9.5
XAF-1407	55.4 ± 1.9	65.3 ± 7.9**	56.7 ± 2.3	60.3 ± 6.7
Path length (cm)				
All	5.2 ± 1.2	8.9 ± 1.7***	7.4 ± 1.1^###^	9.5 ± 1.4***^,#^
Acute AF	—	7.6 ± 0.5	—	7.8 ± 1
Spontaneous termination	—	8.2 ± 0.8	—	9.4 ± 0.7^#^
AP14145	6.3 ± 1.2	9.3 ± 1.7**	8.1 ± 0.9^#^	10.2 ± 1.4**
PA-6	5.8 ± 0.9	8.2 ± 1.2***	7.4 ± 1^#^	9.1 ± 1.2**
Vernakalant	3.9 ± 0.8	9 ± 1.8***	6.6 ± 1.2^###^	9.5 ± 1.5***
XAF-1407	5.2 ± 0.8	9.6 ± 2.1***	7.6 ± 0.8^###^	10 ± 1.1***

### 3.2 Kinetics and dynamics of spatiotemporal organization of AF

**FIGURE 4:**
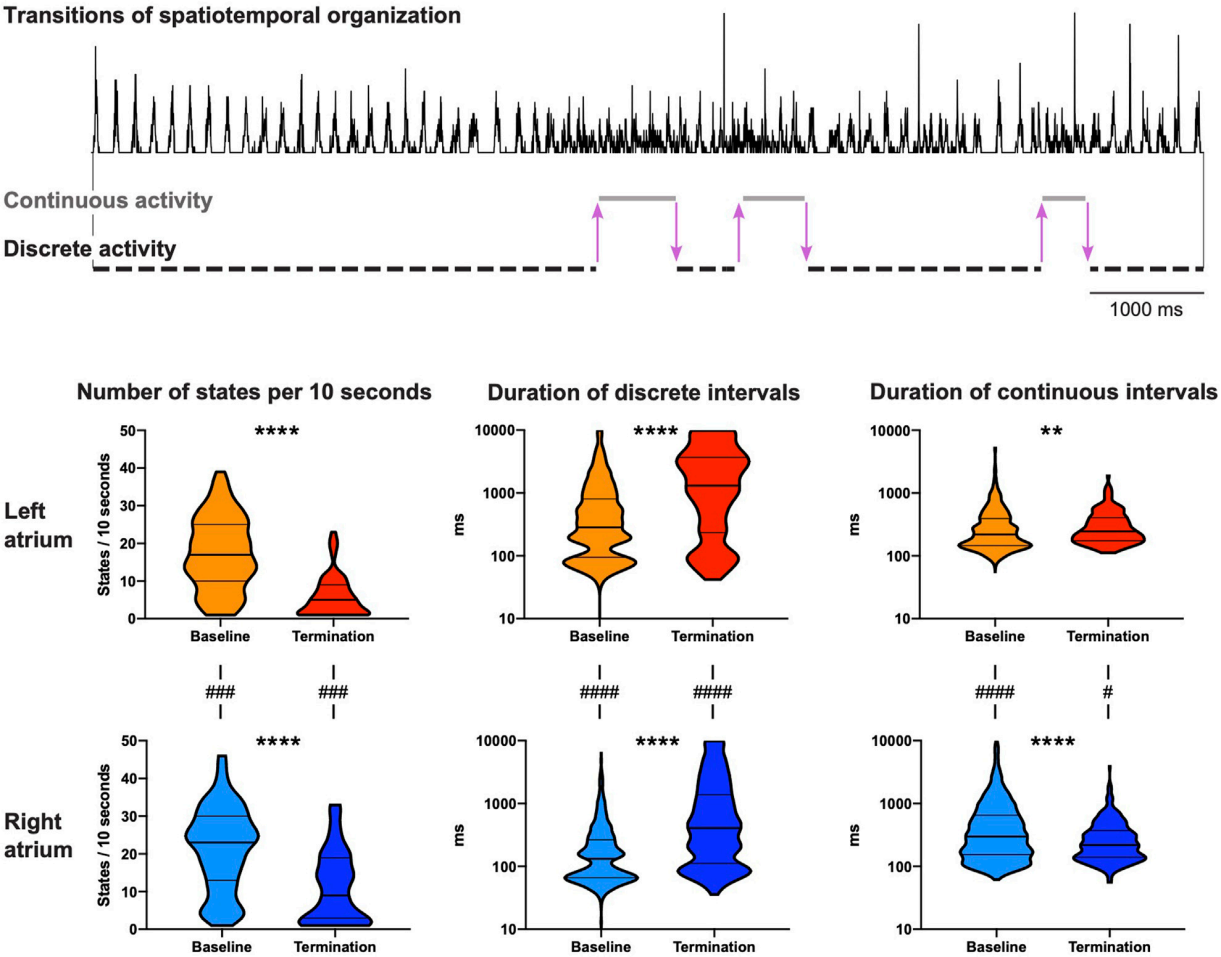
Dynamic properties of spatiotemporal organization. Both continuous and discrete activities were observed at baseline and at 10 s before AF termination. The activation time histogram in the top panel presents an example (PA-6-induced termination) with six transitions in spatiotemporal organization before AF termination in the right atrium (3 transitions from discrete to continuous activity and 3 transitions from continuous to discrete activity, illustrated by purple arrows). Bottom panel shows the number of states (continuous and discrete activity) per 10 s and the duration of continuous and discrete intervals for both atria. Statistical analysis of the differences between baseline and AF termination was performed with a Mann-Whitney *U* test (**p < 0.01, ****p < 0.0001). The comparisons of the number of states between the left and the right atrium were performed with a Wilcoxon test, and for the duration of continuous and discrete intervals with a Mann-Whitney *U* test (^#^ *p* < 0.05, ^###^ *p* < 0.001, ^####^ *p* < 0.0001). 65 AAD-induced AF terminations in 27 animals were included.

During baseline AF, multiple switches between the states of continuous and discrete activity were observed over a 10 s period (Figure 4, top). Electrophysiological changes due to the switches between discrete and continuous activity (LA and RA combined) were limited. AFCL was 1.04-fold (IQR 1.01–1.06) longer, CV 1.10-fold (IQR 1.05–1.14) higher and path length 1.16-fold (IQR 1.09–1.21) longer during discrete activity compared to continuous activity.

**FIGURE 5:**
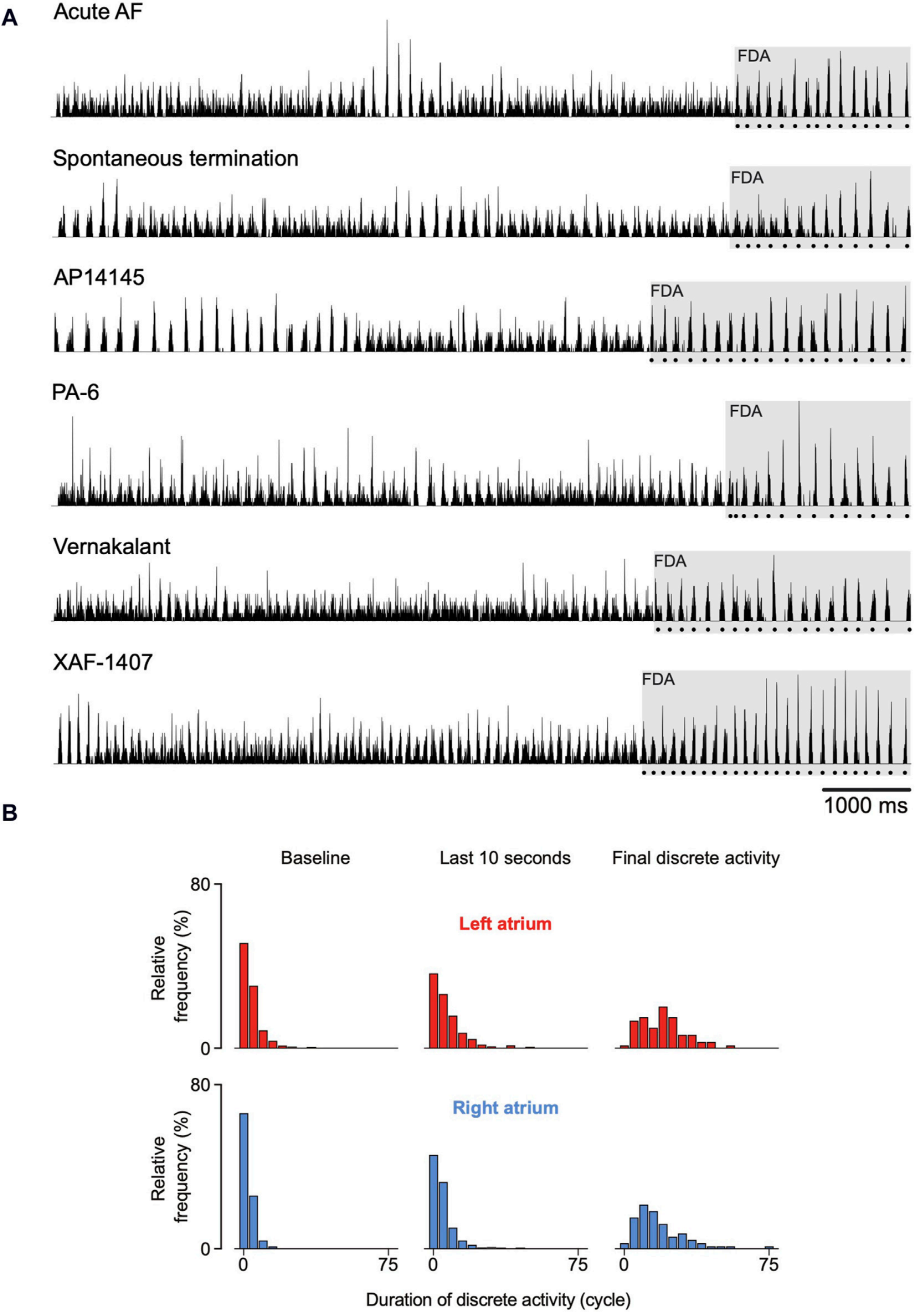
Spatiotemporal organization just before termination of atrial fibrillation (AF). **(A)** Six randomly chosen normalized activation time histograms from the six investigated groups. The dotted line below the histograms delineates the final episode of discrete activity (FDA). **(B)** In general, FDAs lasted more AF cycles than other discrete periods. An FDA could only be detected if at least one transition from continuous to discrete activity occurred during the last 10 s before AF termination. As a consequence, not all terminations could be included and the number of FDAs differed between atria. 64 FDAs were observed in the right atrium and 58 in the left atrium.

The number of states during the last 10 s before termination significantly decreased in both atria (Figure 4, bottom). This occurred mainly due to substantial prolongation of intervals of discrete activity in both atria. As a matter of fact, discrete activity preceded all AF terminations, independent of the degree of continuous activity preceding termination (Figure 5A). In general, this final discrete activity (FDA) persisted for more cycles than the periods of discrete activity that did not result in AF termination (Figure 5B). The number of cycles in the FDA was comparable between the LA and RA and no differences were found in the individual groups (Table 2).

**TABLE 2:** Number of cycles in the final discrete activity. Only the AF terminations containing at least one transition from continuous to discrete activity were considered. The values are reported for individual groups as mean ± SD. No statistically significant differences between the left atrium and the right atrium were found (Wilcoxon test, *p* < 0.05).

	Right atrium		Left atrium	
	n	Cycles	n	Cycles
All	64	15 (10–25)	58	21 (9–26)
Acute AF	8	14 (12–17)	8	13 (8–27)
Spontaneous termination	6	9 (8–18)	4	13 (9–19)
AP14145	12	17 (4–22)	12	24 (22–29)
PA-6	14	13 (10–21)	9	21 (18–35)
Vernakalant	9	19 (15–32)	7	9 (5–22)
XAF-1407	15	16 (9–30)	18	21 (13–22)

### 3.3 Electrophysiological changes during the final episode of discrete activity

**FIGURE 6:**
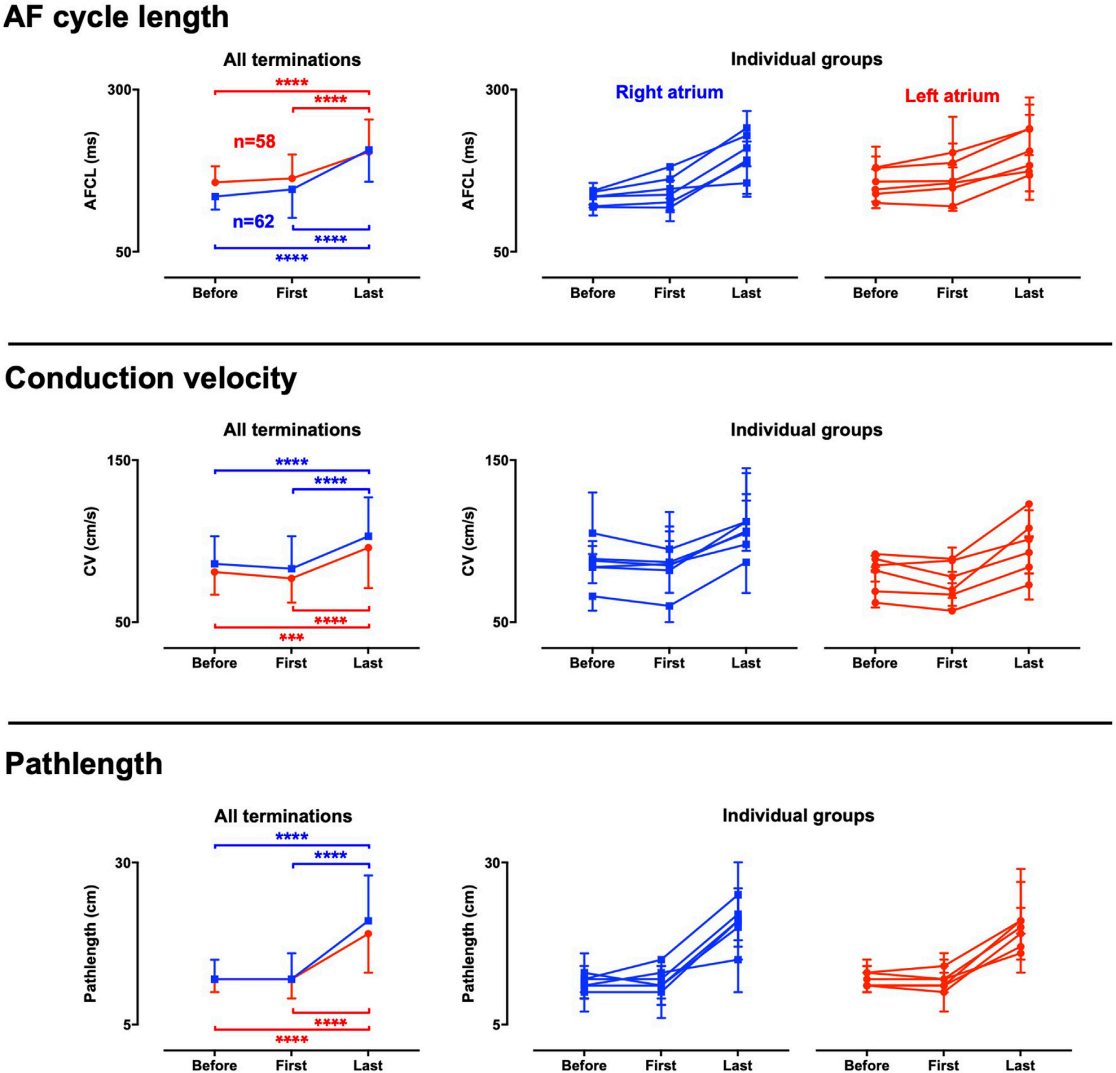
Electrophysiological changes during the final period of discrete activity. Electrophysiological parameters were determined at three critical time points towards AF termination; *Before:* the average of the whole period before the final discrete activity (FDA) occurred, *first:* the first cycle of the FDA and *last:* the final cycle of the FDA. The results for the whole dataset are presented in the left panels. Panels to the right present data for the individual groups, with each line representing a group. An FDA could only be detected if at least one transition from continuous to discrete activity occurred. As a consequence, not all terminations could be included, and the number of FDAs differed between atria. 64 FDAs were observed in the right atrium and 58 in the left atrium. Data were statistically analyzed with a Friedman’s test. ***p < 0.001 ****p < 0.0001.

AF properties particularly changed during the final episode of discrete activity (FDA). Hence, electrophysiological changes occurred on top of the effect of the AAD infusions. To analyze the changes during FDA, the first and last cycle (i.e., the last AF cycle) of the FDA were determined and compared to the average of the preceding period of the last 10 s before AF termination. At the start of an FDA, only a small or no change was observed in the average AFCL, conduction velocity and path length compared to the period leading up to the FDA (Figure 6). However, a robust prolongation of AFCL occurred from the first to the last cycle in the FDA where AFCL became 1.26-fold longer in the LA (IQR 1.07–1.44) and 1.43-fold longer in the RA (IQR 1.27–1.69) in the approximately 20 AF cycles of the FDA. Conduction accelerated during the FDA in all groups, also after initial conduction slowing in case of vernakalant. These phenomena jointly caused a strong path length prolongation. The median path length prolonged 1.74-fold in the LA (IQR 1.20–2.36) and 2.02-fold in the RA (IQR 1.29–2.45) in the approximately 20 AF cycles of the FDA. Similar trend was observed for all groups (Figure 6).

### 3.4 The conduction patterns preceding AF termination

**FIGURE 7:**
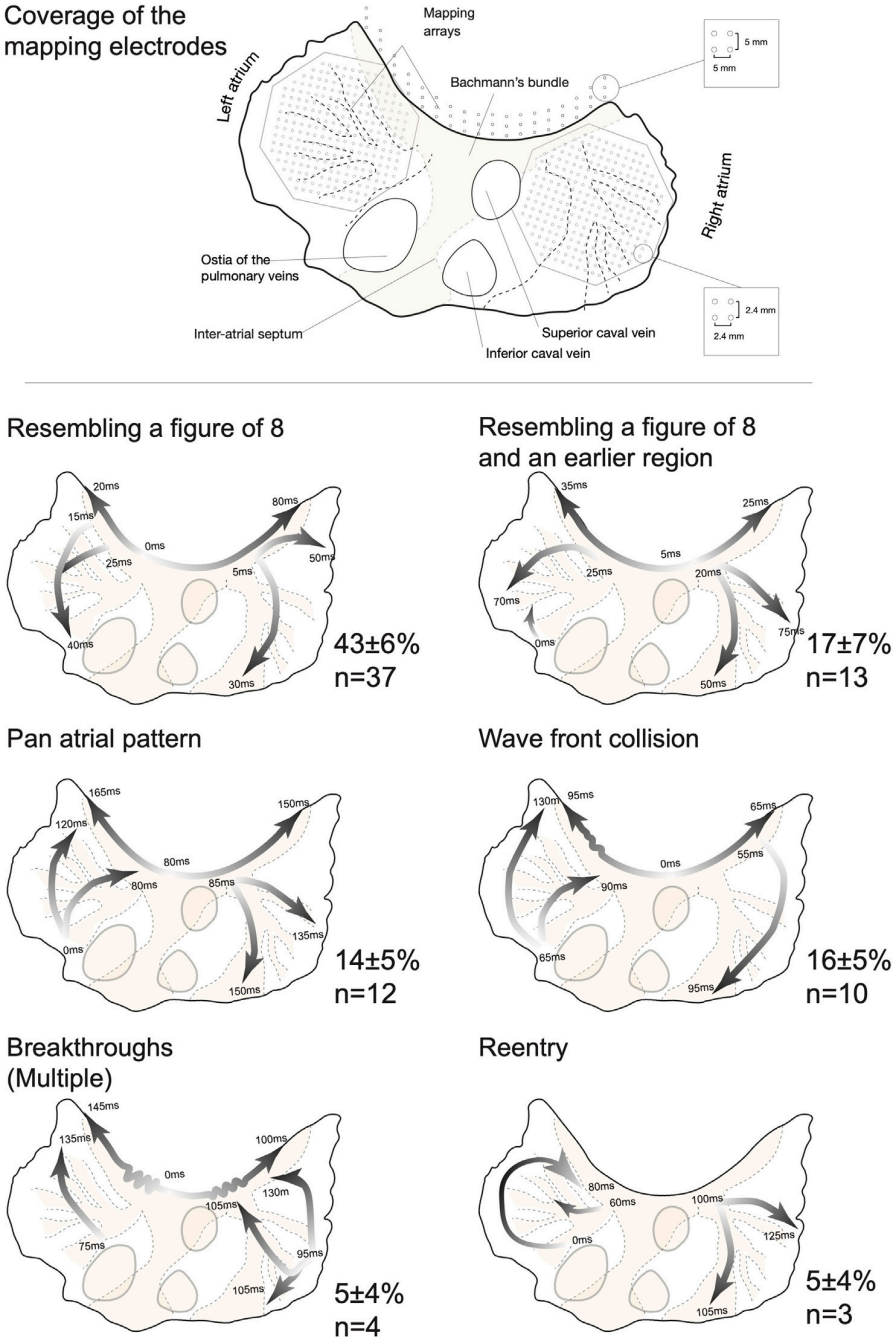
Conduction patterns immediately preceding termination of atrial fibrillation (AF). Top: A topographical outline of the goat atria was created using a specimen that was mounted and transilluminated to visualize the atrial anatomy. Large structures such as the interatrial septum, Bachmann’s bundle and larger bundles are shown by a shade of orange to present the orientation of the mapping electrodes. The Bachmann’s bundle mapping electrode was only present in the vernakalant group. **Bottom:** Examples of the six different types of AF termination patterns. Global wave fronts are presented by arrows, with white shade indicating the start and the dark shade representing the last activated area. The start and end times of the wave fronts are given in milliseconds with the earliest time point being 0 ms. The incidence (in percentage) of patterns per group was determined and averaged (±SEM) for the whole dataset. Table 3 presents a detailed report on the number of observations per group and pattern type.

**FIGURE 8:**
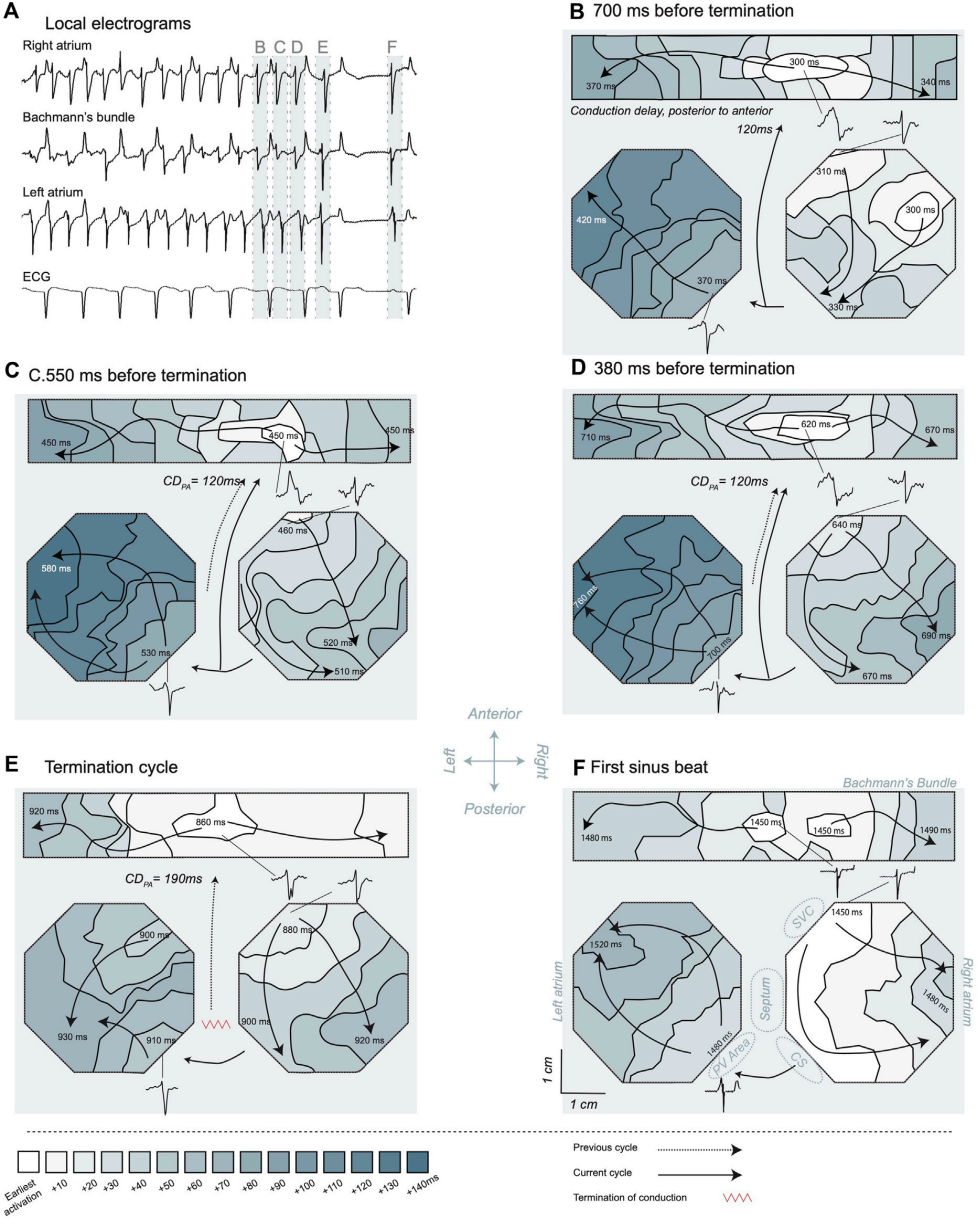
Activation maps of the transition from atrial fibrillation (AF) to sinus rhythm. **(A)** Electrograms recorded in the different mapping areas. The illustrated electrograms are samples of the site of the earliest activation. A long pause after AF termination is visible and the first SR beat resulted in a P-wave in the ECG. The light blue bars and labels B-F highlight the specific time windows corresponding to the maps shown in the subsequent panels. The last four cycles of AF **(B–E)** and the first sinus beat **(F)** are presented, and *t* = 0 ms marked 1 s before termination. For orientation purposes certain larger structures are depicted in panel **(F)**. The posterior to anterior conduction delay was determined based on the last activation time in the preceding activation map and the earliest activation time in the new cycle, in case posterior to anterior conduction was probable. LA, left atrium; RA, right atrium; PV, pulmonary vein; CS, coronary sinus; SVC, superior caval vein. The depicted conduction pattern started ∼700 ms before AF termination **(B)**. Sites of early activation were found in the center of the Bachmann’s bundle and on the anterior side of the RA. Activity over the Bachmann’s bundle propagated towards the RA and LA. Activity in the RA conducted from anterior towards the CS area. About 40 ms after the latest RA activation a wave front started at the posterior side of the LA close to the PV area, suggesting that LA was activated through the posterior connections from the RA. This wave front conducted towards the anterior part of the electrode array. The earliest activation in the second cycle **(C)** was a breakthrough wave in Bachmann’s bundle. The electrogram at this site had an RS morphology, suggesting that an intramural wave front acted as source. Conduction over the septum was the most probable origin of the activation based on the preceding activation map. The conduction delay from posterior to anterior (CD_PA_) took 120 ms. The global pattern was comparable to the preceding beat with anterior to CS conduction in the RA and early activation in the LA at the posterior side close to the PV area. In the penultimate AF cycle **(D)**, again a breakthrough in the Bachmann’s bundle initiated the activation pattern. The global sequence of activation was comparable to the preceding cycles, suggesting that an RA macro-reentrant loop was driving the arrhythmia at this point in time. During the ultimate AF cycle **(E)** the CD_PA_ increased considerably to 190 ms. Furthermore, a shift to a more central location occurred for the breakthrough site on the Bachmann’s bundle. The RA got activated somewhat earlier then the LA and conducted towards the CS. The LA was activated from both the Bachmann’s bundle and at the posterior portion of the LA, resulting in a wave front collision on the LA free wall. During the following 420 ms, the atria remained electrically silent, suggesting that during the final cycle the wave front terminated between the posterior part of the atria and the non-mapped areas, hence in the IAS. The first SR beat **(F)** was characterized by a synchronized earliest activation in the Bachmann’s bundle and on the RA close to the SVC (i.e., from the sinoatrial node), fast conduction from RA to LA and a P-wave in the ECG.

Next, we investigated the global conduction pattern of the final AF cycle to identify the site of arrhythmia extinction. Little variation in termination pattern was observed in the large number of investigated AF terminations. Figure 7 presents examples of all six observed patterns. i) Resembling a figure of 8. Early activation of the Bachmann’s bundle followed by a bifurcation of the wave front. This led to nearly synchronous activation of both atrial free walls, propagating from anterior to posterior (n = 37). Such conduction was reminiscent of a figure-of-eight re-entry with the septum as the common path. This pattern exhibits wave fronts conducting towards the inter-atrial septum (IAS) and pulmonary vein (PV) area, a region that was not covered by the mapping arrays. Yet, the fibrillatory wave fronts did not exit these structures. This implies that termination of propagation of the final fibrillatory wave front(s) occurred at the IAS/PV area resulting in SR. Figure 8 gives a detailed description of such a pattern. ii) Resembling a figure of 8 with an early activated region, often located at the posterior portion of the LA or the inferior portion of the RA (n = 13). This resulted in a collision of wave fronts on the free wall of one atrium. Concurrently, a wave front conducted into the IAS/PV but did not reactivate other atrial structures. iii) A pan-atrial loop formed by dyssynchronous but successive activation of the atria (n = 12). Here, the propagating wave entered the IAS/PV area from a single atrium. Again, the wave front conducted to the IAS/PV area did not exit these structures, implying termination of the wave front propagation at the IAS/PV portion of the atria. iv) Wave front collision, synchronous wave fronts on both atrial free walls but in opposite direction (n = 10). In this case wave front propagation terminated in the Bachmann’s bundle or possibly in the IAS. v) Breakthrough activation of the atrial free walls without an apparent link to the Bachmann’s bundle or the posterior section of the atria (n = 4). No consistent site of termination of wave front propagation could be ascertained. vi) Functional re-entry termination (n = 3). In one case an invading peripheral wave front closed the excitable gap, causing re-entry termination (shown in Figure 7) and the other two cases terminated due to a shortening of the functional block line.

The relative occurrence (in percentage) of these patterns was determined for each group (Table 3) and averaged. Patterns i, ii and iii shared three remarkable features; 1) The Bachmann’s bundle activated at least one atrium, 2) anterior to posterior conduction over the free wall, 3) termination of propagation of the final AF wave front at the IAS/PV area. This specific sequence of events encompassed 77% ± 4% of all terminations.

**TABLE 3:** The frequency distribution of the type of conduction pattern during the final cycle of AF. Six different conduction patterns before AF termination were identified. The number of observations and the relative occurrence (in percentage) of these patterns was determined for each group.

	Figure of 8-like	Figure of 8-like with early activated area	Pan atrial loop	Wave front collision	Multiple breakthroughs	Reentry
All	37	13	12	10	4	3
Acute AF	4 (50)	0 (0)	1 (13)	2 (25)	1 (13)	0 (0)
Spontaneous termination	1 (17)	2 (33)	0 (0)	2 (33)	0 (0)	1 (17)
AP14145	7 (54)	1 (8)	3 (23)	2 (15)	0 (0)	0 (0)
PA-6	8 (57)	1 (7)	3 (21)	2 (14)	0 (0)	0 (0)
Vernakalant	7 (44)	7 (44)	1 (6)	0 (0)	1 (6)	0 (0)
XAF-1407	10 (45)	2 (9)	4 (18)	2 (9)	2 (9)	2 (9)

## 4 Discussion

### 4.1 Main findings

This study investigates the dynamic properties of AF that precede AF termination in goats. The same general pattern leading to AF termination was found both in non-pharmacological (Acute AF group and Spontaneous terminations group) and AAD-induced cardioversions and can be described as follows. Ten seconds before AF termination, a longer AFCL and path length is present compared to baseline AF. These longer cycle lengths led to more frequent episodes of spatiotemporal organization of atrial activity. However, transitions between organized and disorganized activity continue to occur, often until a few seconds before the arrhythmia termination. Eventually, AF always converts to a final episode of spatiotemporal organization. During this phase, a sudden and pronounced AFCL prolongation occurs, coinciding with an acceleration of conduction in atrial free walls. This results in a considerably stronger increase in the path length of fibrillatory waves when compared to the AAD effect that preceded this episode of spatiotemporal organization. During the final AF cycle, the most common conduction path follows preferential propagation over the Bachmann’s bundle, activating the free wall from anterior to posterior and eventually leading to termination of wave front propagation in the IAS/PV area. This finding suggests an important role for atrial anatomy in AF termination.

### 4.2 The transition from AF to SR exhibits consistent electrophysiological patterns

In the current work we investigated non-pharmacological AF terminations and terminations induced by AADs with considerably different current inhibition profiles. Different characteristics of these AADs were intended to translate, at least conceptually, into altered depolarization and repolarization properties. Vernakalant aims for an “early” repolarization and reduced excitability, while AP14145, PA-6 and XAF-1407 should be effective in achieving a delayed “late” phase repolarization. Furthermore, both XAF-1407 and PA-6 should induce a shift in the resting membrane potential. Despite these differences, a consistent prolongation occurred for AFCL and path length, as reported for many other AADs (Workman et al., 2011). These effects gradually built up over a period of many minutes during the AAD infusion (Ji et al., 2017; Gatta et al., 2021; Sobota et al., 2021; van Hunnik et al., 2021). Our study demonstrates that this AAD-induced shift in AF properties alone was insufficient to terminate AF. A larger effect on AFCL and path length occurred for only a couple of seconds during the final episode of stable spatiotemporal organization. This transition occurred both in AAD-treated groups and self-terminating AF, which suggests that the final transition towards SR is a universal process independent of AAD and duration of AF-induced remodeling.

The pronounced AFCL prolongation of this phase can be considered as a fundamental antiarrhythmic mechanism. Short AFCLs are associated with source-sink mismatches in the complex atrial structure, causing spatiotemporal disorganization (Berenfeld et al., 2002). Moreover, AFCL is inversely related to the occurrence of conduction block, number of wave fronts and re-entry (Schuessler et al., 1992). Congruent to these findings, the final episode of AFCL prolongation was associated with simple conduction patterns over the Bachmann’s bundle and both atrial free walls, presumably often leading to block of conduction at the IAS and PV region resulting in termination of AF.

A somewhat surprising observation was the increase in conduction velocity. This observation could be explained by the often-observed activation of the endocardial trabeculated networks via the Bachmann’s bundle, as this would lead to a more synchronized activation of the atrial free walls (van Campenhout et al., 2013). Yet, the strong increase in AFCL is likely to have widened the excitable gap profoundly, as described for other AADs (Wijffels et al., 2000). This widened excitable gap would prolong the time for sodium channels to recover from inactivation, resulting in higher CV. This interaction between AFCL and CV leads to a pronounced lengthening of the path length. Under these conditions, small disturbances of refractory period may lead to conduction block of the final fibrillatory wave.

### 4.3 A shift in the dynamic properties of spatiotemporal organization precedes AF termination

AF is a highly dynamic process with often changing conduction patterns from one cycle to another (Gatta et al., 2021; van Hunnik et al., 2021). In this study we investigated the dynamic properties of spatiotemporal organization of the entire atrial free walls. We showed that frequent fluctuation of spatiotemporal organization in the atrial free walls occurs after 4 weeks of AF in the goat. These data suggest that the fibrillatory process was still inclined to frequently organize to episodes of discrete activity but the AF substrate was advanced to such a degree that the periods of organized activity lasted for only very short time. Yet, longer episodes of discrete activity were required for AF to terminate. The termination process was characterized by prolonged discrete activity during which a robust change in electrophysiological properties, of excessively long path lengths and atrial synchronization, occurred. It would be of interest to study the dynamic behavior of spatiotemporal organization in models with an AF substrate which is resistant to AADs. Under such conditions, the balance between the duration of discrete and continuous activities might be shifted to much longer periods of continuous activity. A robust quantitative description of the dynamic process might be therefore a valuable predictor for the efficacy of AADs under such conditions.

### 4.4 Conduction patterns preceding AF termination match atrial macroscopic structure

The Bachmann’s bundle likely served as an interatrial conduction pathway for the final AF cycle in about 75% of the AF terminations. Moreover, mapping of the Bachmann’s bundle suggested that conduction over the septum initiated the final wave. Both Bachmann’s bundle and IAS are muscular structures that are considered as main pathways of interatrial conduction during SR (van Campenhout et al., 2013; Kharbanda et al., 2019). It is conceivable that AF persistence is facilitated by disturbed conduction in the Bachmann’s bundle and IAS, resulting in dissociation of activity between the atria. Restoring organization of conduction in the Bachmann’s bundle may therefore result in a more coordinated conduction and more synchronized activation of the atrial free walls via the endocardial trabeculated network. Indeed, conduction from the septum to the Bachmann’s bundle often precedes reactivation of the atria during organized AF activity (Kumagai et al., 1997), reminiscent to patterns during the FDA. It may be anticipated that termination of final propagating wave front in the IAS leads to AF termination. For instance, AF inducibility is reduced when the IAS is ablated (Tondo et al., 1997) and full disconnection of the LA from the RA abolishes inducibility of AF (Betts et al., 2001). These observations suggest that during a period of increased organization the conduction over the macro-anatomical structure of the IAS and Bachmann’s bundle becomes critical for AF termination.

### 4.5 Preferential site of arrhythmia extinction

Only a few studies used atrial mapping to describe the process of AF termination. The most detailed investigations were presented in studies by Wang et al. showing that AF termination was often caused by a conduction block in the critical part of a reentrant circuit or a focal site of activation (Wang et al., 1992; Wang et al., 1993; Wang et al., 1994). No preferential site of cholinergic AF termination was reported. In contrast, we observed a tight link between the atrial anatomy and the conduction pattern of termination in acute AF and after 3–4 weeks of AF-induced remodeling in the goat.

The considerably long path length during the final AF cycles approximated the circumference of a goat atrium, often leading to figure-of-eight like patterns. This switch from AF to a flutter like process (Ortiz et al., 1994) sensitizes the arrhythmia perpetuation to a small disturbance of local electrophysiological properties. Our data demonstrate that arrhythmia extinction occurred in about 75% of the AF terminations in the PV and/or IAS region of the atria. The intrinsic electrophysiological properties of the IAS and Bachmann’s bundle, such as faster conduction and longer refractory periods compared to other parts of the atria (van Campenhout et al., 2013; Kharbanda et al., 2019), may favor closure of the excitable gap and re-entry termination. On the other hand, an “external modulator” such as innervation by either sympathetic fibers from the external stellate ganglion or vagal nerve endings that are present in the IAS and pulmonary veins (Gardner and O’Rahilly, 1976) may have acted as a final stride for termination of the macro-reentry or silencing of focal activity.

### 4.6 Clinical perspective

This study demonstrates that increased stability of organized fibrillation is essential for terminating AF. During the presence of organized fibrillation, conduction may progress in a way that leads to excessively long path lengths of fibrillatory waves. These findings suggest that therapies enhancing AF organization and/or promoting bi-atrial activation along the Bachmann’s bundle could increase the likelihood of AF termination. Non-invasive tools, such as body surface mapping or advanced f-wave analysis from a standard ECG, could help to identify patients who naturally experience bi-atrial episodes of organization, are likely to respond to AADs, and are potentially eligible for the “pill-in-the-pocket” strategy. Furthermore, given that we observed similar patterns in both spontaneous and AAD-induced terminations, it can be anticipated that prolonging the AF cycle length is more critical for AF termination than targeting specific ion channels.

### 4.7 Limitations

Mapping with higher atrial coverage would be necessary to unravel the process of AF termination in more detail. Based on our results, mapping the left atrial posterior wall and IAS would be of interest to further unravel the mechanism of AF termination.

## 5 Conclusion

AF termination in goats follows a particular path towards sinus rhythm, independent from the AAD intervention. Before the actual process of AF termination sets in, AF properties have shifted to increased spatiotemporal organization and a longer AFCL, but both remained subject to dynamic changes. The AF termination process is abrupt and can be characterized by stable and high spatiotemporal organization, allowing an additional prolongation of AFCL and acceleration of atrial conduction, resulting in propagation over the IAS and Bachmann’s bundle in the last activation cycle. These findings indicate that AF termination occurs at critical structures of the atrial anatomy.

## Data Availability

The original contributions presented in the study are included in the article/Supplementary Material, further inquiries can be directed to the corresponding author.

## References

[B1] Berenfeld O. Zaitsev A. V. Mironov S. F. Pertsov A. M. Jalife J. (2002). Frequency-dependent breakdown of wave propagation into fibrillatory conduction across the pectinate muscle network in the isolated sheep right atrium. Circ. Res. 90, 1173–1180. 10.1161/01.res.0000022854.95998.5c 12065320

[B2] Betts T. R. Roberts P. R. Morgan J. M. (2001). Feasibility of a left atrial electrical disconnection procedure for atrial fibrillation using transcatheter radiofrequency ablation. J. Cardiovasc Electrophysiol. 12, 1278–1283. 10.1046/j.1540-8167.2001.01278.x 11761416

[B3] Everett IV T. H. Wilson E. E. Foreman S. Olgin J. E. (2005). Mechanisms of ventricular fibrillation in canine models of congestive heart failure and ischemia assessed by *in vivo* noncontact mapping. Circulation 112, 1532–1541. 10.1161/CIRCULATIONAHA.104.521351 16145002 PMC2062530

[B4] Gardner E. O’Rahilly R. (1976). The nerve supply and conducting system of the human heart at the end of the embryonic period proper. J. Anat. 121, 571–587. 1018009 PMC1231747

[B5] Gatta G. Sobota V. Citerni C. Diness J. G. Sørensen U. S. Jespersen T. (2021). Effective termination of atrial fibrillation by SK channel inhibition is associated with a sudden organization of fibrillatory conduction. Europace 23, 1847–1859. 10.1093/europace/euab125 34080619 PMC8576281

[B6] Ji Y. Varkevisser R. Opacic D. Bossu A. Kuiper M. Beekman J. D. M. (2017). The inward rectifier current inhibitor PA-6 terminates atrial fibrillation and does not cause ventricular arrhythmias in goat and dog models. Br. J. Pharmacol. 174, 2576–2590. 10.1111/bph.13869 28542844 PMC5513871

[B7] Kharbanda R. K. Özdemir E. H. Taverne Y. J. H. J. Kik C. Bogers A. J. J. C. de Groot N. M. S. (2019). Current concepts of anatomy, electrophysiology, and therapeutic implications of the interatrial septum. JACC Clin. Electrophysiol. 5, 647–656. 10.1016/j.jacep.2019.04.013 31221350

[B8] Kumagai K. Khrestian C. M. Waldo A. L. (1997). Simultaneous multisite mapping studies during induced atrial fibrillation in the sterile pericarditis model. Insights into the mechanism of its maintenance. Circulation 95, 511–521. 10.1161/01.cir.95.2.511 9008471

[B9] Ortiz J. Niwano S. Abe H. Rudy Y. Johnson N. J. Waldo A. L. (1994). Mapping the conversion of atrial flutter to atrial fibrillation and atrial fibrillation to atrial flutter. Insights into mechanisms. Circ. Res. 74, 882–894. 10.1161/01.res.74.5.882 8156635

[B10] Schotten U. Verheule S. Kirchhof P. Goette A. (2011). Pathophysiological mechanisms of atrial fibrillation: a translational appraisal. Physiol. Rev. 91, 265–325. 10.1152/physrev.00031.2009 21248168

[B11] Schuessler R. B. Grayson T. M. Bromberg B. I. Cox J. L. Boineau J. P. (1992). Cholinergically mediated tachyarrhythmias induced by a single extrastimulus in the isolated canine right atrium. Circ. Res. 71, 1254–1267. 10.1161/01.res.71.5.1254 1394883

[B12] Sobota V. Gatta G. van Hunnik A. van Tuijn I. Kuiper M. Milnes J. (2021). The acetylcholine-activated potassium current inhibitor XAF-1407 terminates persistent atrial fibrillation in goats. Front. Pharmacol. 11, 608410–608413. 10.3389/fphar.2020.608410 33584287 PMC7873360

[B13] Tondo C. Scherlag B. J. Otomo K. Antz M. Patterson E. Arruda M. (1997). Critical atrial site for ablation of pacing-induced atrial fibrillation in the normal dog heart. J. Cardiovasc Electrophysiol. 8, 1255–1265. 10.1111/j.1540-8167.1997.tb01016.x 9395168

[B14] van Campenhout M. J. Yaksh A. Kik C. de Jaegere P. P. Ho S. Y. Allessie M. A. (2013). Bachmann’s bundle: a key player in the development of atrial fibrillation? Circ. Arrhythm. Electrophysiol. 6, 1041–1046. 10.1161/circep.113.000758 24129206

[B15] van Hunnik A. Zeemering S. Podziemski P. Kuklik P. Kuiper M. Verheule S. (2021). Bi-atrial high-density mapping reveals inhibition of wavefront turning and reduction of complex propagation patterns as main antiarrhythmic mechanisms of vernakalant. Europace 23, 1114–1123. 10.1093/europace/euab026 33608723 PMC8286852

[B16] van Hunnik A. Zeemering S. Podziemski P. Simons J. Gatta G. Hannink L. (2018). Stationary atrial fibrillation properties in the goat do not entail stable or recurrent conduction patterns. Front. Physiol. 9, 947. 10.3389/fphys.2018.00947 30100877 PMC6072874

[B17] Wang J. Bourne G. W. Wang Z. Villemaire C. Talajic M. Nattel S. (1993). Comparative mechanisms of antiarrhythmic drug action in experimental atrial fibrillation. Importance of use-dependent effects on refractoriness. Circulation 88, 1030–1044. 10.1161/01.cir.88.3.1030 8353865

[B18] Wang J. Feng J. Nattel S. (1994). Class III antiarrhythmic drug action in experimental atrial fibrillation. Differences in reverse use dependence and effectiveness between d-sotalol and the new antiarrhythmic drug ambasilide. Circulation 90, 2032–2040. 10.1161/01.cir.90.4.2032 7923691

[B19] Wang Z. Pagé P. Nattel S. (1992). Mechanism of flecainide’s antiarrhythmic action in experimental atrial fibrillation. Circ. Res. 71, 271–287. 10.1161/01.res.71.2.271 1628386

[B20] Wijffels M. C. E. F. Dorland R. Mast F. Allessie M. A. (2000). Widening of the excitable gap during pharmacological cardioversion of atrial fibrillation in the goat: effects of cibenzoline, hydroquinidine, flecainide, and d-sotalol. Circulation 102, 260–267. 10.1161/01.CIR.102.2.260 10889140

[B21] Witt C. M. Dalton S. O’Neil S. Ritrivi C. A. Sanders R. Sharma A. (2018). Termination of atrial fibrillation with epicardial cooling in the oblique sinus. JACC Clin. Electrophysiol. 4, 1362–1368. 10.1016/j.jacep.2018.06.016 30336883

[B22] Workman A. J. Smith G. L. Rankin A. C. (2011). Mechanisms of termination and prevention of atrial fibrillation by drug therapy. Pharmacol. Ther. 131, 221–241. 10.1016/j.pharmthera.2011.02.002 21334377 PMC3173850

[B23] Zeemering S. Maesen B. Nijs J. Lau D. H. Granier M. Verheule S. (2012). Automated quantification of atrial fibrillation complexity by probabilistic electrogram analysis and fibrillation wave reconstruction. Conf. Proc. IEEE Eng. Med. Biol. Soc. 2012 2012, 6357–6360. 10.1109/EMBC.2012.6347448 23367383

